# Perspectives on the FDA platform technology designation program for the approval of gene therapies: a Swiss multi-stakeholder exploratory interview study

**DOI:** 10.1186/s13023-025-04038-y

**Published:** 2025-10-14

**Authors:** Yi Han, Kelly E. Ormond

**Affiliations:** 1 Department of Chemistry and Applied Biosciences, Institute of Pharmaceutical Sciences, ETH Zürich, Zürich, Switzerland; 2 Health Ethics & Policy Lab, Department of Health Sciences and Technology, ETH Zürich, Hottingerstrasse 10, 8092 Zürich, Switzerland

**Keywords:** Gene therapy, Advanced therapy medicinal products (ATMP), Platform technology, Rare disease therapy, Drug development, Pharmaceutical regulation

## Abstract

**Background:**

The U.S. Food and Drug Administration (FDA)’s platform technology designation program aims to streamline the development and approval process for advanced therapy medicinal products and is anticipated to be particularly beneficial for the development of gene therapies for rare and ultra-rare diseases. This is an exploratory interview study of Swiss-based perspectives, including insights from stakeholders in industry, academia, regulation, and reimbursement. The objective of our study is to document Swiss professionals’ views on the US platform designation program and to assess the acceptability and feasibility of a similar program in Europe.

**Results:**

Participants identified benefits such as reduced redundancy in pre-clinical testing, standardization of manufacturing, and increased predictability of regulatory requirements. Concerns were raised about clinical assessment, commercialization strategies, and global regulatory alignment. Though participants anticipate it to increase innovation in the rare disease area, some raised the possibility that technologies may stagnate around a platform, or that designations may quickly become obsolete due to the speed of technological development.

**Conclusions:**

The introduction of the platform technology designation program in the US is a step towards increasing treatment options for ultra-rare diseases. While there is potential for platform designation to enable development in this area, its success will depend on addressing the outlined challenges.

**Supplementary Information:**

The online version contains supplementary material available at 10.1186/s13023-025-04038-y.

## Background

There is potential for gene therapies and gene-editing approaches (hereafter referred to as “gene therapies”) to address an area of highly unmet medical need in patients with ultra-rare genetic diseases. A significant challenge to achieving this, however, is their high development and manufacturing costs [1, 2]. The development cost for gene therapies is suggested to be higher than other types of pharmaceuticals due to the use of highly specialized equipment, high-cost reagents, complex patient monitoring during clinical trials, and the use of multiple patent-protected technologies requiring licensing agreements [2]. Manufacturing costs are driven up by personalized supply chains and complex manual manufacturing processes which are difficult to scale up [3, 4]. High costs and low patient numbers limit incentives to develop gene therapies for ultra-rare diseases.

Platform technologies are an array of tools and systems designed to streamline drug production, built around the concept of process standardization [5]. Platforms are widely used in the pharmaceutical industry to simplify drug development and manufacturing, and have been extensively documented in the production of gene therapies [6–11]. Gene therapy platforms are expected to be a critical pathway towards the development and accessibility of bespoke therapies, allowing pharmaceutical companies to streamline a large portion of the research and development and manufacturing process [12, 13]. However, despite these technological advancements, there remains a gap in operationalization of the potential benefits of gene therapy platforms due to lack of standardization in manufacturing methods and regulatory requirements.

The US Food and Drug Administration (FDA) has recognized these barriers and is working to streamline advanced therapy medicinal product (ATMP) development. In December 2023, the US government legalized the Omnibus Appropriations Act of 2023, which includes Section 506 K of the Federal Food, Drug, and Cosmetic Act (21 U.S.C. 356k), requiring the establishment of the Platform Technology Designation Program [14]. Under this program, pharmaceutical companies may apply for platform technology designation for a *“well-understood and reproducible technology […] that is essential to a drug*,* can be adapted for or otherwise used by multiple drugs that share common structural elements*,* and facilitates the manufacturer or development of multiple drugs through a standardized process”* [14]. It would allow market authorization (MA) applications to leverage data from approved products utilizing the same platform technology, reducing evidence requirements and making regulatory requirements more predictable [14]. It is expected to reduce gene therapy development and approval times in particular.

The practical aspects of the platform designation program are still being established. The FDA published a draft guidance document on May 28, 2024, with plans to release a guidance document specifically tailored to gene therapies in 2025 [15, 16]. As of December 2024, we are unaware of any publicly disclosed intentions of the EU or any European countries on the implementation of a similar program. The objective of this study is to document the views held by stakeholders in the Swiss pharmaceutical industry, academia, reimbursement, and regulatory landscapes on the FDA’s program, to inform the potential establishment of platform-based approvals for gene therapies in Europe.

## Methods

We chose an exploratory qualitative approach using semi-structured interviews for Swiss-based participants, serving as a pilot project to future work that will examine attitudes more broadly across Europe. Switzerland was selected for its high concentration of multinational pharmaceutical companies, its strong academic and regulatory presence, and its accessibility to the Swiss-based research team. This study was conducted in compliance with the Swiss Human Research Act, reviewed by the ETH Zürich ethics committee (*EK 2024-N-151*).

### Participant selection and recruitment

The target participant group included experts from the pharmaceutical industry, health regulation or policy, insurance providers, and academic research with positions related to development or market access of gene therapies, or broadly ATMPs in Switzerland. Participants were excluded if they were not either working in Switzerland or employed by a Swiss company at the time of recruitment. Participants were identified via two sampling methods. First, we used a purposive sampling approach, identifying potential participants with publicly available staff profiles online and researchers’ prior professional contacts (*Supplemental Methods*). We followed with a snowball sampling approach; all potential participants, regardless of whether they took part in the interview, were asked to refer additional colleagues both in the recruitment email and after the interviews.

We identified 78 potential participants, 14 of whom could not be contacted due to missing contact information. Between May 21 and September 3, 2024, we sent recruitment invitations to 64 individuals, accompanied by a detailed information and consent document. Of these, 57 (89%) individuals were identified via purposive sampling and 7 (11%) via snowball sampling. The 64 invitees included 26 (41%) from industry, 24 (37%) from academia, 7 (11%) from Swissmedic, and 7 (11%) from insurance providers. Potential participants were preferably contacted via email (75%) but were contacted via LinkedIn messenger (25%) if no public email addresses were found. Two reminder emails were sent approximately 2 and 6 weeks after each initial contact. In total, 45 (70%) individuals did not respond, and 6 (9%) declined due to self-reported lack of expertise on the interview topic.

### Measurement tools and data collection

A novel semi-structured interview guide was created by YH (MSc pharmaceutical sciences) and KO (genetic counsellor, empiric bioethics researcher). The interview guide (*Appendix A*) addressed:

1. The respondents’ opinions about the usefulness of such a program (US, Europe, globally).
2. Potential risks and benefits.
3. Desirability and feasibility in Europe.
4. Barriers and facilitators to implementation in Europe.

Interviews were conducted online via ZOOM by a single interviewer (YH). All interviews were conducted in English to standardize the question format and data analysis. Verbal consent was obtained from all participants prior to conducting the interview. Audio from the interviews was recorded, downloaded, and transcribed via Trint (an AI-powered transcription tool), and the interviewer reviewed and edited transcriptions for accuracy. To ensure participant confidentiality, all interviews were de-identified before data analysis, and all data and contact information were securely stored.

### Data analysis

Coding and data analysis followed a thematic analysis approach in parallel to data collection [17]. The researchers first conducted a *naïve reading* of de-identified transcripts to get an overall understanding of the interviews. Upon the second reading, codes were inductively developed from common themes in the texts. Researchers individually tested the draft codebook on a representative transcript, discussed revisions, then repeated the process twice to achieve the final codebook. Transcripts were coded using the qualitative analysis software NVivo 20. A single researcher (YH) coded all transcripts, with 25% co-coded to consensus by KO to ensure rigor and consistency. Excerpts from each code were analyzed for common themes, which were discussed and validated by both researchers. Quotes were selected for representativeness, and edited for grammar and conciseness while maintaining the content.

## Results

Thirteen semi-structured interviews were conducted from June to August 2024 (20% response rate), from 4 functional areas in pharmaceutical development and market access (Table 1). Interview lengths ranged from 36 to 73 min (median of 51 min). We conducted a sufficient number of interviews to reach thematic saturation, which was defined as the “stage in the data analysis process where repetitive codes or themes are identified, and no new information or relationships between them emerge” [18]. There was one outlier (P07) with unique views that were largely not shared by other respondents.

**Table 1:** Participant demographics and characteristics

Participant characteristics	Number of respondents (%)
Female	5 (38.5%)
Swiss citizen	7 (53.8%)
Age (years)	30–54 (mean = 43.8, median = 47)
Roles	
Industry, Supporting	4 (30.8%)
Industry, Drug development	3 (23.1%)
Academia, Drug development	3 (23.1%)
Academia, Other	1 (7.7%)
Government regulatory	1 (7.7%)
Insurance provider	1 (7.7%)
Experience in this area (years)	1–30 (mean = 12.7, median = 12)
Total	**13**

Two participants stated that they were unaware of the platform designation program prior to being invited to participate in this study. None of the participants had direct experience with the FDA platform designation program. One participant stated that their group is considering pursuing platform designation, and two participants stated that they know other groups pursuing platform designation. Along with their professional expertise, two participants were themselves a patient or caregiver for a patient with a rare genetic disease, and brought both perspectives to the discussion. 

Four significant themes were identified in the data: (1) definitions of a “platform” vary, (2) potential implications of platform designation, (3) “it sounds simple, but it’s quite difficult to execute”, (4) regional differences.

### Definitions of a “platform” vary

When asked to define a platform, most participants spoke about platform in the context of gene therapies. Respondents primarily gave examples of gene therapies for genetic diseases rather than oncology indications. Participants often referred to the platform technology as the delivery mechanism (Table 2), mentioning specific technologies (e.g. viral capsids, lipid nanoparticles, RNA sequences, and antisense oligonucleotides). Some responses included promoters or the editing technique. Occasionally, respondents gave use cases that did not focus on gene therapies, such as vaccines, targeted chemotherapy delivery systems, hematopoietic stem cell therapies, bispecific or monoclonal antibodies, RNA therapies, and CAR-T cells.

**Table 2:** Representative quotes for theme 1

Definitions of a “platform” vary
Representative quotes
*“Basically*,* these platform approaches share a common technology*,* a common pharmacokinetics*,* a common pharmacodynamics. They can be characterized by a certain number of assays.”* (P04 – Academia, Drug development) *“You could have a library of different capsids that have certain tropisms. And then you have promoters that are already used and approved. […] And then*,* you would just adjust the payload. Then the next level you say*,* ‘okay*,* it’s a similar disease. We know the promoter works*,* but with a very different payload*,*’ and that’s stretching the platform concept a bit further. And then of course*,* you can go like*,* ‘well*,* the platform is just the capsid’ […] and then you add different elements to it.”* (P01 – Industry, Drug development) *“People understand the definition of platform in different ways. We’re viewing our device as a platform for manufacturing multiple different therapies. In my read of the recently released FDA guidance on platform regulation*,* it was more about the therapeutic side. You have a method of delivering a cargo and that’s your platform. […] You’ve qualified your delivery method so that’s much closer to the current biology*,* but it doesn’t address ‘how do we do the manufacturing?’”* (P13 – Industry, Supporting)

Beyond the technology integrated into the product, some participants also felt that the manufacturing technology required to produce it was an important aspect of a “platform” (Table 2). Some expressed dissatisfaction with the limited definition of platform technologies in the legislation, proposing the inclusion of platforms as medical devices, drug-device combos, and manufacturing platforms. When asked about programs with similar characteristics to the platform designation program, respondents likened it to the biomarker qualification program, agnostic labels, and the hospital exemption scheme in Europe.

### Potential implications of platform designation

Participants suggested that the platform designation program could impact a wide range of processes in R&D, manufacturing, market access, and commercialization. Participants agreed that the platform designation program was created with the intention to improve patient access, accelerate the development of new therapies, and increase innovation especially in the area of ultra-rare diseases (Table 3). However, there was uncertainty about its effect on innovation, how the pace of innovation might influence outcomes, and potential impacts on safety and efficacy.

**Table 3:** Sub-themes and representative quotes for theme 2

Potential implications of platform designation	
Sub-themes	Representative quotes
Usefulness of the program	*“The very first platform technology we are using is the flu vaccine. You know the seasonal updates. And I think it might be fashionable to create new regulations on platform technologies*,* especially in the field of mRNA*,* to name just one. But this is more likely to be a PR stunt than anything else*,* because technically we already can regulate platforms in Switzerland*,* in Europe*,* and all over the place. So the need to change the regulation is not that huge in the eyes of a regulator.”* (P07 – Government regulatory)
Improving patient access	*“The general caution that we have right now when it comes to cell and gene therapy must be changed a little bit. Should be less. Because for some kinds of diseases*,* there’s actually no other choice than a gene therapy. Look at SMA [Spinal muscular atrophy]*,* look at Duchenne muscular dystrophy. There are conservative therapies maybe*,* but people still die. So the only chance that you have might be such a radical therapy like gene therapy.”* (P02 – Industry, Supporting) *“This is really moving away from the concept of platform technologies*,* because now we go in directions where there’s even a smaller population of patients and more complex technologies*,* which often require very specific designs and engineering. And I think we will have a situation where the needs of bringing these technologies to patients will be very different from the technical needs adapted to the development of novel technologies.”* (P11 – Academia, Drug development)
Accelerating drug development	*“The amount of effort required for an ultra-rare disease*,* to develop something from scratch to do all of the preclinical clinical work from scratch*,* is just huge. And it would cost several tens of millions. But if you could minimize that to essentially verification of what has been done before*,* and you can go directly into a patient study that is well observed*,* that would make a huge difference.”* (P01 – Industry, Drug development) *“The issue is that even though often the same backbone is being used*,* (so really the same vector type*,* for example*,* AAV9*,* AAV5)*,* sponsors really have not been able to leverage what’s known about the platform itself. And so they have to undertake a lot of testing*,* which is in a way redundant. And so then results in a loss of time*,* but also wastage*,* and particularly animal wastage*,* because of the fact that the biologically sensitive species predominantly for gene therapies is the non-human primate.”* (P09 – Industry, Drug development) *“I think we have this time of the Valley of Death in the early development phase. And I think this is where a platform approach would have the biggest impact.”* (P11 – Academia, Drug development) *“They would probably shorten all clinical phases if they still are all needed. I would expect to have other forms than classical III or IV phases. […] So you would probably do something like a phase I/II and then a confirmatory phase III*,* but with the same size as a normal phase II. […] I don’t think there is a stage that is unaffected in normal clinical development. Depending on the stability of the platform*,* phase IV may not exist at all*,* or may be intensified as commitments in order to show that the platform really performs as it should.”* (P07 – Government regulatory)
Impact on manufacturing	*“I think the big challenge*,* from a drug development standpoint*,* is around manufacturing CMC. There still aren’t standards for some of the analytics in process development*,* […] so sponsors are coming with all different ways of doing this. I think the FDA really wants to try and get to a standardization of that. But my sense is in Europe*,* they’re even further behind in terms of coming up with standards for CMC development.”* (P09 – Industry, Drug development) *“Manufacturing is a major risk because […] the costs associated with manufacturing are such that it is difficult to really have multiple opportunities. Viral vector technologies are difficult to manufacture because you have to start from scratch every time. […] And if now you say*,* ‘okay*,* we have this vector technology which is now accepted as a platform*,* and the manufacturing is de-risked.’ These may really have a major impact for investors because that’s exactly the type of questions they ask you. ‘What’s the level of risk?’”* (P11 – Academia, Drug development)
The effect on innovation is uncertain	*“If you are kind of forcing people to be more efficient*,* then that could drive innovation. If on the other hand*,* you say 'you don’t need to bother with that*,* just reference prior product applications*,'* then there’s a risk that you dampen innovation.”* (P09 – Industry, Drug development) *“You want to have the best promoter for that patient setting. You want to have the best capsid. So if you can improve the capsid further and you get more payload into a difficult to reach tissue*,* you would want to do that*,* so then you have to leave the platform anyways. So I do believe there is also a natural limitation to like*,* you can come up with something better. I would rather use the better treatment.”* (P01 – Industry, Drug development) *“If you have a platform technology*,* you may end up in a situation where you will realize that maybe it’s not the perfect tool that you have*,* but at least it will help you to get access to the patients*,* because it will allow you to go through this hurdle of having to develop a treatment that otherwise would be too expensive.”* (P11 – Academia, Drug development)
Safety and efficacy concerns	*“I would say that lowering the barrier to entry is not necessarily a hindrance to patient safety. I think the way that it’s done now is*,* especially in the EU*,* probably a bit too stringent. […] It’s not necessarily going to negatively affect patient safety*,* but it’s also not necessarily going to preserve you from it.”* (P03 – Industry, Drug development) *“If you can show … that the process of manufacturing is stable*,* that you can control CPPs*,* there’s no concern in terms of safety […] Doesn’t matter where you produce it and doesn’t matter if you’re using column A or column B*,* because this platform is shown to be safe. […] You can say*,* this platform is safe regardless of the kind of mutation that I’m inserting in a gene therapy that I’m working with. […] The background information like if this CRISPR-Cas therapy is dangerous has already been tested*,* has already been characterized and the answer is already there.”* (P02 – Industry, Supporting) *“I would be very skeptical to actually say*,* ‘well*,* I will give you an easier approval just because the delivery method is the same.’ Because I think the delivery method is secondary to the mechanism of action of the inserted gene. That is what makes the potential side effects and the potential therapeutic effects. […] If the delivery method can safely address that issue*,* then yes*,* it might give an advantage. […] It is also not clear whether there can be any immune reactions. It could be that the efficacy weans off. The gene gets diluted again.”* (P08 – Insurance provider) *“Even with the same capsid*,* you get significantly different expression results in tissue with different promoters or different payloads. […] There are protein sequences that express less well. There might be some folding issues. Typically also*,* the protein needs to integrate into the cellular system. So*,* even if it is somehow expressed*,* but the fold does not support the integration*,* for example*,* in the membrane*,* you will have severe issues to show the right expression where it should happen.”* (P01 – Industry, Drug development)

#### Usefulness of the program

Two respondents questioned whether a platform designation would add value given the existing ability to leverage prior data. They gave examples of products that have already had reduced regulatory load to gain market authorization and wished for clarification on the difference between gaining approval via leveraging prior data vs. platform designation. Outside of the context of gene therapies for genetic diseases, multiple participants said that platform designation would likely be useful for targeted delivery in oncology, and one respondent mentioned that it may be useful for pandemic situations.

#### Improving patient access

Discussions around benefits of platform designation often focused on reducing barriers in drug development for ultra-rare diseases. By reducing development costs, platform designation could increase the likelihood for more solutions to be pursued. Though patient safety was an important consideration, respondents generally wished to prioritize treating patients as quickly as possible. Some respondents mentioned that platform designation would create the opportunity to develop treatments for heterogeneous genetic diseases, which cannot be treated with a single gene therapy. However, one respondent argued that the heterogeneity of ultra-rare indications may require increasingly specific engineering. Beyond development, participants said that platform designation would make the review process more predictable, reducing uncertainty for applicants and administrative burden for regulators.

#### Accelerating drug development

Participants were optimistic that platform designation would allow time savings in all stages of development. Most responses focused on the benefits to preclinical and manufacturing, with only some mentioning benefits in clinical. Many respondents believe that platform designation will have the biggest impact on the late-stage transition from the preclinical to clinical phase. Participants involved in gene therapy development spoke of current redundant requirements for potency, biodistribution, and toxicology assays for previously characterized vectors, which not only has negative implications for the speed and cost of development, but also raises ethical concerns around redundant animal experiments. Two respondents quoted possible time savings of 1 to 2 years per product in the preclinical phase. Others stated that the time savings would depend on the product, or that the specifications and requirements of the program must be fully established before making such predictions.

Participants suggested that having prior clinical knowledge of the platform would allow investigators to develop more informed study protocols, enhancing trial design efficiency; for example, facilitating a better risk assessment before moving into clinical studies. Some participants suggested that platform designation may shorten clinical trial phases or require fewer patients to be enrolled, while others believe that it would be difficult to streamline the clinical phase. Participants were divided on whether post-market study requirements would be reduced or intensified.

#### Impact on manufacturing

Many respondents stated that manufacturing is one of the greatest challenges for gene therapies. Developing a gene therapy manufacturing pipeline is considered extremely risky due to the complex and costly process, and certificates of analysis are not standardized. Because there is no consensus on how to conduct quality assays, each sponsor must develop them separately. Having a standard manufacturing platform would improve manufacturing efficiency in multiple ways: (1) sponsors would not need to develop an entirely new manufacturing process for each product, (2) it would vastly speed up or eliminate the need to develop new quality assays for each new product, (3) it may enable more than one product to be manufactured in the same space.

#### The effect on innovation is uncertain

The majority of respondents were optimistic that increased innovation would be the main long-term benefit of platform designation, listing multiple ways that it may increase innovation: (1) it would allow companies to focus on innovating around the platform, (2) it may allow resources to be routed into other development projects, increasing innovation elsewhere, (3) having alternative routes to authorization supports attractiveness to do research and to bring new products to market. However, some were concerned that relying on platform technologies would cause technological advancement to stagnate if companies prioritize getting products to the market instead of developing more effective delivery methods. There were also worries that granting platform designation to an immature technology would create compounding negative effects on future technologies based on that platform. A separate issue was that platform designations may quickly become obsolete due to the speed of technological development. However, some participants suggested that even outdated platforms can still be valuable by enabling development that would otherwise be prohibitively expensive.

#### Safety and efficacy concerns

When addressing safety concerns, participants first acknowledged the severe and potentially long-term side effects resulting from sequence-specific toxicities associated with gene therapies. There was disagreement about how platform designation would affect product safety. Many participants believe that it would have little effect on safety, or trust that the program would be implemented in a way that adequately addresses safety. Some argue that platform approaches may not have a significant impact on safety even if there are reduced clinical data requirements, because the amount of data that can be collected for ultra-rare diseases is already limited, and gaps can be addressed with post-marketing surveillance. Others believe that it could improve safety. For example, clinical safety data from the platformed products could be pooled, increasing the potential sample size in rare disease areas. Additionally, the development of a standard manufacturing platform could allow companies to better optimize manufacturing processes and standardize quality assays between products, improving safety within a platform. Finally, some respondents were concerned about safety risks introduced by platform designation because the major safety risks originate from the gene sequence, and not the delivery mechanism.

Efficacy was another critical consideration. Participants described prior cases of gene therapies with unstable expression levels due to gene dilution. There were concerns about the inability to re-dose most gene therapies in cases of insufficient efficacy. This has ethical implications for clinical trial participants and patients, and increases the significance of ensuring sustained therapeutic efficacy. Participants suggested that efficacy would likely be unaffected by standardizing the delivery mechanism with a platform because it depends largely on the expression level of the therapeutic gene insert. Even with the same delivery mechanism, different sequences may exhibit differences in delivery, expression levels, and stability over time.

### “It sounds simple, but it’s quite difficult to execute”

#### Assessment of platform products

Operationalizing the assessment of platform products was a major point of concern for participants involved in gene therapy development. Though there were differing views on how a platform approach would affect safety and efficacy, participants were in agreement that assessing the effects of sequence-specific changes introduces challenges that have complex, indeterminate solutions (Table 4).

**Table 4:** Sub-themes and representative quotes for theme 3

“It sounds simple, but it’s quite difficult to execute”	
Sub-themes	Representative quotes
Assessment of platform products	*“It will be very difficult to say*,* based on post-hoc analysis due to low statistical power*,* that there is a certain class of patients that were negatively impacted by the compound. Let’s say*,* for whatever reason*,* that creates an off-target effect*,* an unwanted sequence specific effect. How will you even discover this in clinical development? […] What if you have multiple things*,* and one thing among them is really making it hard? To even discover that*,* I think it’s very difficult. So patient safety is the biggest concern with platform approaches. […] Lack of efficacy can come out from a pooled product*,* whereas individual products would be successful on their own. Very simple example*,* let’s say*,* if every mutation leads to a different speed of degeneration*,* and you make a big class of them. Some of them have the time to make the change*,* and others not*,* so on average*,* you might not see a difference and the whole platform would fall apart. […] So showing efficacy with platform approaches is very risky unless your effect is dramatic.”* (P04 – Academia, Drug development) *“What preclinical study would you need to do to show similar expression levels? And then also you need to experience what are the expression levels that the certain disease manifestation requires from a protein of interest*,* to make it a platform approach. Having said that*,* it sounds simple*,* but it’s quite difficult if you want to execute on that”* (P01 – Industry, Drug development)
Implications on commercialization strategies	*“In manufacturing you will have an unbalanced cost of goods sold. So if in one mutation you have more patients*,* for the other one*,* you have only two. The manufacturing cost is the same. So your profits are dramatically different.”* (P04 – Academia, Drug development) *“If your next therapy to come out is receiving its approval because it’s in the chain of that platform*,* it will be very difficult to adjust the pricing out of the reference frame of the initial or the subsequent early approved therapies of that same platform. […] Effectively*,* the first approval would set the ceiling*,* and all others you would expect are going to be priced lower. […] You then lose a commercial incentive to bring your therapy through.”* (P13 – Industry, Supporting) *“In case the data they generate is also used to make it easier for other companies*,* they might see that as a competitive disadvantage because they already invested all the time and money to prove that some vector is safe*,* and others benefit from it. So maybe that could be an issue.”* (P10 – Academia, Drug development) *“Often you would have a situation where you have a patent holder*,* and they licensed it out to a lot of different companies. […] If one of them says*,* ‘Okay*,* we can use it for many different diseases*,* so we get a platform designation*,*’ what happens then with the others? […] If I’m not the patent holder*,* if I have a non-exclusive license and then obtain a platform designation*,* and if I grant rights of reference to this platform designation to other companies*,* how does this interfere with my patent license?”* (P12 – Academia, Other)

New issues arise from the need to assess a potentially large number of platform products. Participants pointed out that each mutation in a heterogeneous genetic disease may have a slightly different phenotype, and it is important to first understand their clinical manifestations in order to establish meaningful efficacy endpoints. Study parameters may need to be changed for different mutations (e.g. using a different dosage or follow-up times for each product), and the number of clinical assays conducted may be very high depending on the number of mutations targeted. One participant suggested that though platform designation may be facilitative of mixed-population umbrella trials, that approach may result in a statistical lack of efficacy. Finally, participants were concerned that detection of a safety signal from one product on a platform may cause a company to discard the entire platform, which could manifest in both clinical and post-marketing studies.

#### Implications on commercialization strategies

The commercialization strategy for platform products was raised in multiple interviews. Participants said that large pharmaceutical companies are less likely to see a feasible commercial strategy in pursuing platform designation because the types of products that might benefit from platform designation do not align with their business models. There was uncertainty around how different products on a single platform would be expected to be priced, given that development and manufacturing costs would remain consistent across all products using that platform. Another disincentive emerges wherein companies may be hesitant to become the “first innovator”; making the upfront investment to prove clinical efficacy of a platform technology, while future products based on their technology see better commercial success due to improved manufacturing methods. For this reason, participants were concerned about the extent platform designations would be published by the FDA. Other implementation factors may largely impact the commercial incentive to pursue platform designation, such as right of reference and interaction with patent licenses.

### Regional differences

#### The US sets the stage

Participants involved in drug development noted that sponsors tend to favor the US for research and development because it offers better research funding and more flexible regulatory pathways compared to the EU (Table 5). They added that companies prioritize gaining MA in the US due to its large population, free drug pricing, higher risk tolerance, and lack of delay from reimbursement review. The EU offers a comparable market size, but the EMA is seen as more conservative, and reimbursement negotiations must be conducted in each country. Some participants expressed dissatisfaction with the stringency of approvals in Europe, while others maintained that the level of caution is warranted. Some participants said that there is high potential for development in Switzerland due to the amount of innovation and strong push towards new technologies, but difficulties arise due to restrictions on non-human primate studies, few gene therapy experts, and higher cost of clinical research.

**Table 5:** Sub-themes and representative quotes for theme 4

Regional differences	
Sub-themes	Representative quotes
The US sets the stage	*“The US dominates the R&D and drug development world*,* because they have easier regulatory pathways. And we’re kind of just shooting ourselves in the foot by making things harder. […] It’s basically impossible to receive an animal license to do monkey work here. […] And all of this stuff really hinders the development process here. […] But I think this does then impact the need or the appetite for a kind of platform regulation*,* because if you don’t have local research in the preclinical world then you lose the need for platform approvals in translating from preclinical to clinical. And why is that? Because if everything ends up happening in the US*,* […] the US is not going to come to Switzerland to approve things here.”* (P03 – Industry, Drug development) *“FDA is much more about*,* ‘take the risk and then mitigate and monitor*,*’ vs. a European approach*,* which is ‘prove that it’s all okay. And then we will say it’s okay’ And they struggle with*,* ‘what’s the line that you’ve proven enough?’ And there’s always more that you could do and always more you could prove. But then you’re delaying again and again and again*,* the access to the therapy.”* (P13 – Industry, Supporting) *“There are very bad recent examples of FDA approvals in gene therapies for medications that do not show an effect. Europe and Switzerland had a very different stance*,* looking at more the facts. […] I think the [EMA] is excellent. It’s a little bit slower*,* but sometimes it’s a good thing. The FDA sometimes has this stance that*,* ‘okay*,* let’s put it on the market and then we will do post-marketing studies.’ And companies are not interested in the post-marketing studies because they know it’s not going to work that well*,* but in the meantime they sell. And this philosophy is not here in Europe or Switzerland.”* (P04 – Academia, Drug development)
Implementation of a platform program in Europe	*“I think if it comes from the US*,* then I think it will take some time ‘till it’s adopted in Europe. […] And Europe is much more conservative. Not conservative*,* but maybe*,* pricing is much more a governmental affair*,* where all the technology*,* the drugs are assessed in a different way. Maybe in a more systematic way. So I think I could imagine that it’s probably adopted less fast in Europe.”* (P08 – Insurance provider) *“I think the problem really is — the most important pharma market where you have two thirds of all pharma sales are just in the United States. And if we do something now in Europe or in Switzerland*,* how does this affect things globally? I mean*,* do companies care about this or is it just another thing that you might take into account*,* but doesn’t really matter for the pharma industry?”* (P12 – Academia, Other)
“The best would be if this program works worldwide”	*“The best would be if this program works worldwide*,* harmonized among the different authorities. Because to set standards for the US and set standards for Switzerland*,* and standards for the EMA*,* that’s not good for Europe. It’s not going to be the way forward*,* because then you have three types of standards. […] If you are not able to set up a worldwide program*,* then at least there should be alignment of a faster recognition for platform technologies*,* so that you don’t waste time. […] Otherwise*,* the aim of faster availability of those kinds of drugs to everybody is only a local thing.”* (P02 – Industry, Supporting) *“ATMPs target a lot of orphan*,* rare diseases or genetic diseases that are not that widespread. If you need to make too much work*,* and it costs too much money*,* and it takes too much time to expand your therapy to the rest of the world*,* then for sure this is going to bring delay*,* […] which is a drawback to what this program is trying to promote.”* (P02 – Industry, Supporting)

#### Implementation of a platform program in Europe

Most participants believe that a platform designation program will likely be implemented in the EU. However, regardless of their level of support, participants suggested that the EU should observe results in the US before considering implementation. The speed of implementation in the EU would depend on its impact in the US. Participants gave ranges from 5 to 10 years (P02) to 20–30 years (P12), with consideration for the time to develop multiple platformed products in the US.

Participants explained that Switzerland exhibits aspects of an attractive market, being a relatively wealthy country in which multiple large pharmaceutical companies are based. Despite these advantages, there are few gene therapies authorized in Switzerland compared to the US or EU. Because the Swiss market is not a priority, the complex regulatory framework and effort required to establish a similar program would have an uncertain benefit. As a compromise, participants suggested that Switzerland could instead acknowledge FDA or European Commission (EC) approvals for platformed products.

#### “The best would be if this program works worldwide”

Throughout interviews, respondents emphasized the importance of global harmonization of requirements, increased communication, cooperation, and acknowledgement of other regulators’ work. Multiple respondents said that lack of regional and global alignment is one of the biggest hurdles for implementation and usefulness of the platform designation program. There must be standardization and transparency of requirements between regions to avoid requests for redundant clinical testing.

## Discussion

The data from this study captures Swiss-based perspectives from stakeholders in ATMP development, regulation, and reimbursement, who would be most affected by the FDA platform technology designation program. While acknowledging the potential to increase innovation and patient access in the rare disease space, there were uncertainties about what the program would entail, and what tradeoffs may ensue. At a basic level, given the current ability to leverage prior data, some participants were uncertain whether the platform designation program provides any additional value to the current development and approval processes. Others raised technical complexities, suggesting that the practical implementation and utilization of platform designation may be more difficult than anticipated for both regulatory bodies and pharmaceutical companies. Finally, a major concern was the operationalization of the program, especially with respect to reference rights and publication of designations, which would heavily influence its perceived value. At the time of writing, there is no published literature examining the impact of this program on drug development in the US.

In discussing the concept of a “platform,” participants referenced varying interpretations. Historically in the industry, a “platform” typically refers to a wide range of tools, including technologies used for screening, characterization, or manufacturing, but definitions may differ between companies [19, 20]. The definition provided in the legislation is narrower than this classical idea, focusing on the technology directly incorporated into drug products. This divergence was felt strongly in some interviews, with participants speaking of their idea of a “platform” as both the incorporated technologies and manufacturing technologies, while the FDA platform technology definition only applies to the former. International regulators, including from the EMA and MHRA, have also expressed dissatisfaction with the legislation’s limited definition, with many hoping that the program would encompass manufacturing platforms [21].

Though they were not previously referred to as “platform designations,” approaches that leverage prior data, such as those used for flu vaccines and cancer immunotherapies, are globally well-established. In the US, the ability to reference prior data is permitted under FDA regulation 21 CFR § 314.54, which enables the use of data from studies of similar drugs, published research, or previously approved drugs [22]. The EC similarly offers the Hybrid Marketing Authorization Application pathway under Directive 2001/83/EC [23]. One significant limitation is that platform designation will only be granted for technologies that have been incorporated in a previously approved product, which would limit eligibility to the select few entities with gene therapies on the market [14]. Supporting companies, such as those designing vectors but are not drug manufacturers themselves, therefore cannot apply for platform designation, meaning commonly used vectors may not be able to receive designation as participants had hoped. With this taken into account, there is uncertainty about the additional value provided by platform designation, and participants who recognized parallels with the existing regulation were less enthusiastic about the program. In particular, P07 working in regulation saw little added benefit to the platform designation program. They anticipate that there would be little difference in the amount or type of prior data required to achieve regulatory approval, whether it is submitted through the existing pathway or via platform designation. Similar concerns were discussed in an interview with a representative from the FDA’s Center for Biologics Evaluation and Research (CBER) at the 2024 CASSS WCBP conference, who confirmed that while the concept of leveraging data is not new, platform designation would formalize and increase the predictability of regulatory requirements [24]. Further investigation into how platform designation might bring unique benefits to current practices is needed.

The primary aim of the platform designation program is to increase efficiency in the development of ATMPs with a particular focus on supporting rare disease gene therapy development [15, 16, 25]. Participants recognized potential benefits of platform designation that align with existing literature, including time and cost savings during pre-clinical testing, standardization of manufacturing and review, and the ability to address ultra-rare and heterogeneous genetic diseases [11–13, 26, 27]. Beyond what has been previously outlined, participants also suggested that clinical studies could be streamlined by increased bridging of trial phases or reducing patient enrolment requirements. On a larger scale, through global harmonization, participants hope that platform designation will increase access equity between countries, especially those with less mature regulatory infrastructure. A potential benefit not addressed in our data is that each product on a platform would increase process understanding, allowing continuous process improvement [28]. Having platform designation would then allow manufacturing improvements to be applied across all products linked to a platform, eliminating the burden of filing changes for each individual product [25].

Participants also discussed challenges such as clinical assessment strategies, commercialization issues, and disincentives for development. To the best of our knowledge, these criticisms have not been expressed in any publicly available literature. Multiple participants in our study worried that complexities in clinical testing could significantly impact development (the practicality of safety and efficacy assessment for a large array of products on a platform). Paradoxically, these concerns may be especially difficult to overcome for heterogeneous genetic diseases, for which participants said platform designation would facilitate development. Not only would different mutations introduce heterogeneity in the study population, but patient characteristics may already be very diverse due to the small number of patients with these rare indications, further complicating clinical assessment [29]. Classical challenges for small population clinical trials, such as population heterogeneity, recruitment difficulties, and poorly understood natural history, may persist or be amplified by platform approaches [29]. It remains to be seen whether these challenges pose a sufficient disincentive to pursue development for such disease areas. While participants expressed some concerns about safety, many noted that the risk threshold is often higher for rare or severe diseases. This aligns with the views of patients and caregivers of patients with rare diseases, who are often willing to accept higher risks or side effects for novel treatments [30]. These patients are often put into a position where they must consider turning to off-label or high-risk experimental treatments due to the lack of well-established treatment options [31]. Despite its challenges, the platform designation program may facilitate the pursuit of innovative approaches to address these unmet medical needs.

Due to technical complexity and high costs of gene therapy development and manufacturing, and other factors such as hesitancy around reimbursement, an appropriate commercial model may be difficult to develop for platform-based products. Currently, rare disease development is mainly being pursued by small to medium-sized companies [32–34]. Participants suggested that large pharmaceutical companies view platform designation unfavorably, given their existing reluctance to invest in rare disease development [35, 36]. Further, while the legislation restricts the ability to leverage platform designation to its owner and those holding reference rights, participants had concerns about whether, and to what extent, designations will be published; a concern that has also been raised by others in the field [37]. Even without reference rights, competing companies could draw on publicly available information about a platform to inform their own R&D. This issue may be particularly relevant for biologics where some components cannot be patent protected. Additionally, the interaction of patent licenses and platform designations remains unclear. Outstanding questions include whether, and to what extent, platform designation applications can reference patented technologies from other companies, and what types of agreements are needed to do so.

The prevailing assumption, in both our data and existing literature [5, 12, 38], is that the use of platform technologies should reduce development costs. However, a notable counterpoint argues that the use of platforms may ultimately have little impact on overall drug development spending [39]. The costs associated with establishing and maintaining a platform are substantial even when distributed across multiple drug development projects. Meanwhile, the rate of new drug approvals continues to decline regardless of increased spending on platform technologies [40]. Unfortunately, accurately estimating the financial impact of platforms is challenging, especially for large pharmaceutical companies managing multiple concurrent projects.

Finally, while all participants in this study were based in Switzerland, discussions largely centered around the US and EU due to their status as the most significant global markets. In 2022, North America and Europe accounted for 52.3% and 22.4% of global pharmaceutical sales respectively [41]. The US and Europe continue to dominate in pharmaceutical R&D, with expenditures growing annually. Though there are many other factors influencing patient access in different regions, prioritization of these two regions from development to marketing contributes significantly to why other countries lack or have later access to new products [42–44]. Participants suggested that it may be more beneficial for Switzerland to recognize authorization of platform products granted in other countries rather than establishing a dedicated regulatory framework. The impact of not establishing a similar program would depend on how accepting Swissmedic is of platform-based approvals from other countries. This approach could benefit other countries with less attractive markets, helping to achieve greater patient access to ATMPs beyond a select few large, wealthy countries.

## Limitations

While this study generated meaningful perspectives, some limitations may impact the conclusions drawn. First, as the study was restricted to Swiss-based participants, findings may reflect a predominantly Swiss-centric perspective. Second, self-selection bias may have favoured recruitment of participants with more polarized views on the platform designation program. Third, the participant distribution was uneven, with greater representation from industry and academia, and only one participant each from regulation and reimbursement. It is possible that the full scope of perspectives within these two groups was not captured. While our sample size is on the lower end of what is typically seen in a qualitative study, we expect the limitations to be minimal, since data collection continued until thematic saturation was reached. Finally, because this topic is so closely tied to industrial strategies, much of the relevant research about platform technologies is likely confined to proprietary or grey literature, therefore limiting the availability of prior studies to inform our discussion.

## Conclusions

The introduction of the platform technology designation program in the US is a step towards increasing treatment options for ultra-rare diseases. For the program to have a meaningful impact on global patient access, there must be harmonization of regulatory requirements and mutual acceptance of prior data between different health authorities. Exploring ways to further incentivize rare disease research for large pharmaceutical companies could also broaden the impact of the platform designation program. Future research should involve global stakeholders alongside US and European perspectives to ensure that the potential impacts of this program are considered while it is still in early stages.

## Supplementary Information

Below is the link to the electronic supplementary material.

Supplementary Methods

## Data Availability

The transcripts used and/or analysed during the current study are available from the corresponding author upon reasonable request.

## Appendix

Detailed interview guide

This interview is being done as part of a research project conducted by the Health Ethics and Policy Lab at ETH Zurich. It will be about platform approvals for the market authorization of gene therapies.

You were recruited because you have experience in either development or market access of ATMPs and gene therapies in Switzerland. We’ve chosen an interview format specifically so we can hear your detailed thoughts about the topic, so please feel free to elaborate on your responses.

Anything you say in your interview that might identify you (such as your name or workplace) will be removed from the transcript before data analysis. If our findings are published in a poster or a journal article, you would be referred to as, for example, “Respondent 3 – Industry, Drug development” if we use any direct quotes from you.

If you’re worried about anonymity, I would be happy to reach out to you again when we have a draft before we submit it anywhere for publication. Would you like us to check in with you at that time?

Finally, do you have any questions before we start the interview?

Confirm consent to record their responses.

1. Descriptive info

Firstly, we would like an idea of your professional role in pharmaceuticals, to provide context for your responses.

- Could you give me an overview of what you currently do for work? In general, not only related to the interview topic.

- *Prompt* general role or involvement in: regulation, policy, HTA, pricing/reimbursement, insurance, industry, research, clinic/patient care.
- *Prompt* what does it involve?

- How long have you worked in ____/in these roles?

- Do you have previous experience in any other area of market access?

- In what country do you work currently?
- Have you worked in a related role in any other countries?

- Which ones?

For our demographics statistics, would you be able to tell us:

- Age
- Gender
- Swiss citizen? (yes/no)

2. Your experience with gene therapies

Now we want to know how your work specifically relates to the market access of gene therapies.

- *(Regulatory, policy)* How are you currently involved with the market authorization of gene therapies in Switzerland?
- *(Industry, clinicians, HTA, insurance)* How does gene therapy authorization regulation impact you or your work?

- *Prompt* for example, if a GT is approved/not approved…

- How long has your work been involved with gene therapies/ATMPs?

- *Prompt* Can you give me an example of…
- *Prompt* Can you tell me more about it…
- *Prompt* What are some of the worries people have expressed about GT so far in your experience?

Let's pivot to touch on the main topic of this interview. The FDA has recently launched a “platform designation program” for the market authorization of drugs that have the same or similar manufacturing methods (December of last year). We want to make sure everyone’s on the same page and has the same definition for a platform technology.

By the FDA definition, a platform technology is a well-understood and reproducible technology that is incorporated or used by a drug, is essential to the structure or function of that drug, and can facilitate the manufacture or development of more than one drug product.

Confirm that aligns with interviewee’s understanding.

A company can apply for platform designation on a specific platform technology, and once they receive that designation, they might have fewer or more streamlined regulatory requirements for subsequent drugs using that platform.

We’re interested to know the sentiments of the key stakeholders (people in the pharmaceutical industry, from regulatory bodies, healthcare payers, clinicians) on the idea of implementing a similar platform designation program, mostly focusing on Switzerland in this interview. If you have the expertise, we would also be interested to know if you have any input more broadly in Europe as well.

3. What do you know about platform designation in the US

- I sent the FDA legislation document via email. It’s okay if you didn’t read that in detail, but would you be able to explain what you know about the platform designation program in the US, in your own words?
- Do you have any experience working with the platform designation program in the US?
- Are you aware of any similar programs already existing in Switzerland (and/or Europe), for any class of drugs (not just gene therapies)?

- IF YES ask for opinions about them.

- Are you aware of any sentiments held by others in the field about platform approvals of gene therapies in Switzerland (and/or Europe)?

- IF YES Are there any vocal advocates or critics for implementing platform approvals in Switzerland and/or Europe?

- Are you aware of any movements in the Swiss and/or European regulatory scene related to the implementation of a platform designation program or something similar?

4. Usefulness of platform designation program

So now we want to know your opinions about the platform designation program.

- In general, what are your opinions or feelings on the platform designation program in the US?

- *Prompt* can you elaborate? What do you mean by ___?

If positive:

- Do you think it would be useful to implement a similar program in Switzerland and/or Europe?

- How/why would it be useful? in what contexts?
- Should it be implemented?

- How/why would it be useful? in what contexts?
- Should it be implemented?

- Would there be any differences in the type, or degree of benefit a platform designation program would have in the US vs Switzerland and/or Europe?

- If they don’t understand the question: would it be equally useful in all the same ways in …

- Are there any ways that it could be considered more useful in either country?

- How & why?

- What types of products do you think this program is most useful for?

- Are there any other types of products that might utilize this program to gain market authorization?

- What are some potential benefits that you can see in implementing this program in Switzerland and/or Europe?

- How & why?

- Which stages in getting a product to the market would a platform designation program benefit?

- How & why?
- *Prompt* can you see it benefiting any other step in the route to market access? e.g. for development, manufacturing, authorization, pricing/reimbursement (if not mentioned)
- *Prompt* Do you have an idea or estimate of how much time it would be able to save in the development period?

5. Potential negative impacts

If negative

- Can you think of any reasons that a platform designation program might negatively affect

- drug regulation (all drugs)
- patient safety
- drug innovation
- any other aspect

Before we move on did you have any other thoughts on the potential benefits and harms of platform approvals?

6. Implementation: Barriers and Facilitators

Next, I’d like to touch on potential barriers and facilitators to implementation in Switzerland and/or Europe.

- What might hinder the implementation of a similar program in Switzerland and/or Europe?
- Can you think of any stakeholders in Switzerland and/or Europe who would oppose a platform designation program?
- Can you think of any stakeholders who would be facilitators or would especially support it?

7. Consequences of implementation/no implementation

- Which downstream processes after market authorization might be affected by platform approvals?

- *If they don’t understand the question* referring to HTA, pricing regulation, insurance coverage, patient access.
- *Prompt* How do you think gaining authorization via a platform approval will affect HTA evaluations of gene therapies?
- *Prompt* “ pricing and reimbursement decisions of gene therapies

- Can you think of anything that might result if the US has this program but Switzerland and/or Europe doesn’t?
- How likely do you see a regulation like this being passed in the near future in Switzerland and/or Europe?

- When & why?
- Ask for elaboration

Before we move on did you have any other thoughts on what we just talked about around barriers/facilitators to access, and consequences of implementation or no implementation?

8. Final thoughts—gene therapy regulation

To conclude the interview, let's depart from platform designations to get your final thoughts on gene therapy regulation in general

- How does the regulatory scene surrounding gene therapies in Switzerland compare to that of other countries?

- to Europe?
- to the US?

- Do you have an opinion on what is the most important or pressing challenge for the market/patient access of gene therapies right now?
- That’s all of the questions I have for you today. Do you have any final thoughts that we haven’t already covered?

## References

[1] Marks P, Witten C. Toward a new framework for the development of individualized therapies. Gene Ther. 2021;28:615–7. 32242078 10.1038/s41434-020-0143-y PMC8598985

[2] Sabatini MT, Chalmers M. The cost of biotech innovation: exploring research and development costs of cell and gene therapies. Pharmaceut Med. 2023;37:365–75. 37286928 10.1007/s40290-023-00480-0

[3] Moutsatsou P, Ochs J, Schmitt RH, Hewitt CJ, Hanga MP. Automation in cell and gene therapy manufacturing: from past to future. Biotechnol Lett. 2019;41:1245–53. 31541330 10.1007/s10529-019-02732-z PMC6811377

[4] Shupe J, Zhang A, Odenwelder DC, Dobrowsky T. Gene therapy: challenges in cell culture scale-up. Curr Opin Biotechnol. 2022;75:102721. 35398708 10.1016/j.copbio.2022.102721

[5] Niazi SK. The united States food and drug administration’s platform technology designation to expedite the development of drugs. Pharmaceutics. 2024;16:918. 39065616 10.3390/pharmaceutics16070918 PMC11279857

[6] Felberbaum RS. The baculovirus expression vector system: A commercial manufacturing platform for viral vaccines and gene therapy vectors. Biotechnol J. 2015;10:702–14. 25800821 10.1002/biot.201400438 PMC7159335

[7] Collins LT, Beatty W, Moyo B, Alves-Bezerra M, Hurley A, Lagor W et al. Covalently linked adenovirus-AAV complexes as a novel platform technology for gene therapy. 2024.

[8] Bulcha JT, Wang Y, Ma H, Tai PWL, Gao G. Viral vector platforms within the gene therapy landscape. Signal Transduct Target Ther. 2021;6:53. 33558455 10.1038/s41392-021-00487-6 PMC7868676

[9] Wang D, Tai PWL, Gao G. Adeno-associated virus vector as a platform for gene therapy delivery. Nat Rev Drug Discov. 2019;18:358–78. 30710128 10.1038/s41573-019-0012-9 PMC6927556

[10] Turgeman G, Pittman D, Müller D, Gowda Kurkalli R, Zhou B, Pelled S. Engineered human mesenchymal stem cells: a novel platform for skeletal cell mediated gene therapy. J Gene Med. 2001;3:240–51. 11437329 10.1002/1521-2254(200105/06)3:3<240::AID-JGM181>3.0.CO;2-A

[11] Brooks PJ, Ottinger EA, Portero D, Lomash RM, Alimardanov A, Terse P, et al. The platform vector gene therapies project: increasing the efficiency of Adeno-Associated virus gene therapy clinical trial startup. Hum Gene Ther. 2020;31:1034–42. 32993373 10.1089/hum.2020.259 PMC7585601

[12] Mackall CL, Bollard CM, Goodman N, Carr C, Gardner R, Rouce R, et al. Enhancing pediatric access to cell and gene therapies. Nat Med. 2024;30:1836–46. 38886624 10.1038/s41591-024-03035-1

[13] Nilapwar S, Sousa M, Tscheschke B, Gomez S, Garrett K, Stebbins M et al. Leveraging platform and process characterization data to accelerate CGT validation and commercialization. 2024 Feb.

[14] U.S. Congress. Platform technologies. Nov, 2024.

[15] Center for Drug Evaluation and Research. Center for Biologics Evaluation and Research. Platform Technology Designation Program for Drug Development Guidance Document. 2024 May.

[16] Marks P, Barkek R, Bens C. Alliance Webinar Budget Series: Webinar Transcript of CBER Director Dr. Peter Marks. 2024.

[17] Naeem M, Ozuem W, Howell K, Ranfagni S. A Step-by-Step process of thematic analysis to develop a conceptual model in qualitative research. Int J Qual Methods. 2023;22.

[18] Rahimi S, khatooni M. Saturation in qualitative research: an evolutionary concept analysis. Int J Nurs Stud Adv. 2024;6:100174. 38746797 10.1016/j.ijnsa.2024.100174 PMC11080421

[19] Lewis K. Platforms for antibiotic discovery. Nat Rev Drug Discov. 2013;12:371–87. 23629505 10.1038/nrd3975

[20] Welch AR. Two Spicy Takeaways On The FDA’s Platform Designation Guidance [Internet]. Advancing RNA. 2024 [cited 2024 Nov 16]. Available from: https://www.advancingrna.com/doc/two-spicy-takeaways-on-the-fda-s-platform-designation-guidance-0001

[21] Cerullo M, Zhang Y. The fda’s platform provision: an emerging debate among regulators. Advancing RNA; 2024.

[22] U.S. Food and drug Administration. Procedure for submission of a 505(b)(2) application requiring investigations for approval of a new indication for, or other change from, a listed drug. U.S. Congress; Nov, 2024.

[23] European Parliament and Council. Directive 2001/83/EC of the European Parliament and of the Council of 6 November 2001 on the Community code relating to medicinal products for human use [Internet]. 2022. Available from: https://eur-lex.europa.eu/legal-content/EN/TXT/?uri=CELEX%3A32001L0083

[24] Eglovitch JS. Pharma industry questions FDA on platform technology designation program [Internet]. Regulatory Focus. 2024 [cited 2024 Nov 16]. Available from: https://www.raps.org/News-and-Articles/News-Articles/2024/1/Pharma-industry-questions-FDA-on-platform-technolo

[25] Welch AR, Peter Marks. An Early Preview of mRNA, Gene Therapy Platforms [Internet]. cell&gene. 2023 [cited 2024 Nov 16]. Available from: https://www.cellandgene.com/doc/peter-marks-an-early-preview-of-mrna-gene-therapy-platforms-0001

[26] Buchman G. Getting Gene Therapy into the Fast Lane [Internet]. the Medicine Maker. 2024 [cited 2024 Nov 16]. Available from: https://themedicinemaker.com/manufacture/accelerating-gene-therapy-manufacture

[27] Armstrong A. FDA rolls out platform technology guidance to smooth future regulatory encounters [Internet]. Fierce Biotech. 2024 [cited 2024 Nov 16]. Available from: https://www.fiercebiotech.com/biotech/fda-rolls-out-platform-technology-guidance-smooth-future-regulatory-encounters

[28] Siedler M. Implementation of a Platform Approach for Early Biologics Development [Internet]. Am Pharm Rev. 2011 [cited 2024 Nov 16]. Available from: https://www.americanpharmaceuticalreview.com/Featured-Articles/37325-Implementation-of-a-Platform-Approach-for-Early-Biologics-Development/

[29] Day S, Jonker AH, Lau LPL, Hilgers R-D, Irony I, Larsson K, et al. Recommendations for the design of small population clinical trials. Orphanet J Rare Dis. 2018;13:195. 30400970 10.1186/s13023-018-0931-2 PMC6219020

[30] Morel T, Aymé S, Cassiman D, Simoens S, Morgan M, Vandebroek M. Quantifying benefit-risk preferences for new medicines in rare disease patients and caregivers. Orphanet J Rare Dis. 2016;11:70. 27225337 10.1186/s13023-016-0444-9 PMC4881055

[31] Kesselheim AS, McGraw S, Thompson L, O’Keefe K, Gagne JJ. Development and use of new therapeutics for rare diseases: views from patients, Caregivers, and Advocates. The patient - Patient-Centered Outcomes Res. 2015;8:75–84. 10.1007/s40271-014-0096-6 25362528

[32] Ekins S, Wood J. Incentives for starting small companies focused on rare and neglected diseases. Pharm Res. 2016;33:809–15. 26666772 10.1007/s11095-015-1841-9 PMC4777685

[33] Farkas AM, Mariz S, Stoyanova-Beninska V, Celis P, Vamvakas S, Larsson K et al. Advanced therapy medicinal products for rare diseases: state of play of incentives supporting development in Europe. Front Med (Lausanne). 2017;4. 10.3389/fmed.2017.00053 PMC5432638 28560211

[34] Rollet P, Lemoine A, Dunoyer M. Sustainable rare diseases business and drug access: no time for misconceptions. Orphanet J Rare Dis. 2013;8:109. 23879976 10.1186/1750-1172-8-109 PMC3735431

[35] Seoane-Vazquez E, Rodriguez-Monguio R, Szeinbach SL, Visaria J. Incentives for orphan drug research and development in the united States. Orphanet J Rare Dis. 2008;3:33. 19087348 10.1186/1750-1172-3-33 PMC2631478

[36] Oliveira P, Zejnilovic L, Canhão H, von Hippel E. Innovation by patients with rare diseases and chronic needs. Orphanet J Rare Dis. 2015;10:41. 25887544 10.1186/s13023-015-0257-2 PMC4404234

[37] Church R, Druckman M, Fadahunsi Y, Walsh B, Grey A. FDA Platform Technology Designation Program aims to speed development of drugs, biological products [Internet]. Hogan Lovells. 2024 [cited 2024 Nov 16]. Available from: https://www.hoganlovells.com/en/publications/fda-platform-technology-designation-program-aims-to-speed-development-of-drugs-and-biological-products#:~:text=The%20U.S.%20Food%20and%20Drug,summarize%20FDA’s%20newly%2Dclarified%20criteria

[38] Labant M. Platform processes May reduce AAV gene therapy costs. Genetic Eng Biotechnol News. 2024;44:42–4.

[39] Grainger D. Industry Voices: Platform technologies–The foundations of Big Pharma or its nemesis? [Internet]. Fierce Biotech. 2013 [cited 2024 Nov 16]. Available from: https://www.fiercebiotech.com/r-d/industry-voices-platform-technologies-foundations-of-big-pharma-or-its-nemesis

[40] Platform Tech For Cell &. Gene Therapy Processing Scale-Up [Internet]. World Pharma Today. 2024 [cited 2024 Nov 16]. Available from: https://www.worldpharmatoday.com/news/platform-tech-for-cell-gene-therapy-processing-scale-up/

[41] EFPIA. The Pharmaceutical Industry in Figures. 2023.

[42] Wilsdon T, Armstrong H, Sablek A, Cheng P. Factors affecting the location of biopharmaceutical investments and implications for European policy priorities. London; 2022 Oct.

[43] World Health Organization. Access to medicines: making market forces serve the poor. 2017.

[44] Yonemori K, Hirakawa A, Ando M, Hirata T, Yunokawa M, Shimizu C, et al. The notorious drug lag for oncology drugs in Japan. Invest New Drugs. 2011;29:706–12. 21286780 10.1007/s10637-011-9638-0

