# Supplementary material for: Perspectives on the FDA platform technology designation program for the approval of gene therapies: a Swiss multi-stakeholder exploratory interview study
Source: Orphanet J Rare Dis. 2025 Oct 14;20:514. doi: 10.1186/s13023-025-04038-y (PMC12522643; doi:10.1186/s13023-025-04038-y)
Supplement: Supplementary file 1 — Supplementary Methods [file 13023_2025_4038_MOESM1_ESM.docx]

# ***Supplemental methods: Participant selection***

We conducted a systematic online search using Google.com as the search engine. We aimed to identify Swiss stakeholders with relevant expertise in gene therapies or broadly ATMPs from pharmaceutical companies, supportive industries, academic institutions, insurance providers, and Swissmedic. We searched company websites and LinkedIn to identify and obtain contact information for individuals in senior or influential roles.

**Pharmaceutical companies**

We identified companies with operations in Switzerland that develop or manufacture products related to gene therapies, including supporting industries such as developers of gene therapy production technologies and consultancies. No exclusion criteria were applied based on company size, age, or disease focus.

**Academic institutions**

We searched department websites of major Swiss research universities to identify research groups currently working in gene therapy development.

**Regulatory agency**

Swissmedic staff were identified via the agency’s website and LinkedIn profiles.

**Health insurers**

Swiss health insurance providers were identified via company websites and online directories. Staff were identified via company websites and LinkedIn profiles.

**Selection of stakeholders**

Participants were selected based on their professional seniority and decision-making influence within their organizations. Categories included:

- Chief officers (*e.g.*, Chief Scientific Officer, Chief Medical Officer)
- Directors, Senior Vice Presidents, global or regional heads
- Senior scientists with leading research responsibilities
- Members of expert panels or advisory boards

These categories were chosen because individuals in these roles are most likely to have strategic oversight, policy influence, or technical expertise relevant to gene therapy development, regulation, and/or reimbursement. The same selection criteria were applied to prior contacts. No exclusion criteria were applied based on years of experience or role type. Whenever possible, all potential participants were contacted, including multiple individuals working in the same company or in similar roles.
